# Sleep Disturbance and Subsequent Suicidal Behaviors in Preadolescence

**DOI:** 10.1001/jamanetworkopen.2024.33734

**Published:** 2024-09-16

**Authors:** Joshua L. Gowin, Joel Stoddard, Ted K. Doykos, Mary D. Sammel, Rebecca A. Bernert

**Affiliations:** 1 Department of Radiology, University of Colorado Anschutz Medical Campus, Aurora; 2 Department of Psychiatry, University of Colorado Anschutz Medical Campus, Aurora; 3 Children’s Hospital Colorado, Aurora; 4 Department of Biostatistics and Informatics, Colorado School of Public Health, University of Colorado Anschutz Medical Campus, Aurora; 5 Department of Psychiatry and Behavioral Sciences, Stanford University School of Medicine, Stanford, California; 6 Stanford Medicine, Stanford Health Care, Stanford, California

## Abstract

**Importance:**

Suicide is a leading cause of death among adolescents, who demonstrate high rates of sleep disturbance. Poor sleep appears to confer risk for suicide, but longitudinal investigation of suicidal behaviors remains rare, particularly in the transition from childhood to early adolescence.

**Objective:**

To evaluate sleep disturbances in preadolescent children (aged 9 and 10 years) in association with longitudinal risk for suicidal ideation and suicide attempts at the 2-year follow-up.

**Design, Setting, and Participants:**

This cohort study used data from the Adolescent Brain Cognitive Development Study between June 2016 and January 2021. This dataset included children aged 9 or 10 years at baseline and their parents or caregivers who were recruited at 21 sites across the US. Data were analyzed July 2023 to June 2024.

**Exposures:**

The Sleep Disturbance Scale for Children, a 26-item parent-reported inventory, was administered at baseline, generating a total score and 6 subscales. Central covariates included the Child Behavior Checklist Anxiety and Depression subscale and demographic variables.

**Main Outcomes and Measures:**

The computerized Kiddie Schedule for Affective Disorders and Schizophrenia (K-SADS-COMP) assessed parent- and youth-reported suicidal behaviors and outcomes (none; passive, active nonspecific, and active specific suicidal ideation; and suicide attempt) at the 2-year follow-up. Sleep disturbance was further grouped by symptom severity (minimal, moderate, elevated, high, and severe).

**Results:**

Of the 10 136 youths who reported no baseline suicidal ideation or behavior, 8807 youths (mean [SD] age, 9.9 [0.6] years; 4507 males [51.2%]; 197 Asian individuals [2.2%], 1273 Black individuals [14.5%], and 5775 White individuals [65.6%]) completed the K-SADS-COMP assessment at the 2-year follow-up and were included in the analysis. At the follow-up, 8044 participants (91.3%) had no suicidal behavior, 317 (3.6%) had passive suicidal ideation, 258 (2.9%) had active nonspecific suicidal ideation, 130 (1.5%) had active specific suicidal ideation, and 58 (0.7%) had a first-time suicide attempt. Baseline sleep disturbance was associated with increased incidence risk for suicidal behavior at age 12 years (odds ratio, 2.68; 95% CI, 1.44-4.98; *P* = .002), adjusting for covariates. Individual subscales or items demonstrating associations with risk included disorders of excessive somnolence and frequency of nightmares.

**Conclusions and Relevance:**

Results of this longitudinal cohort study revealed that disturbed sleep at age 10 years was associated with risk for suicidal thoughts and behaviors in the next 2 years. The findings highlight the potential importance of sleep as a visible risk factor and intervention target in the prevention of youth suicide.

## Key Points

**Question:**

What is the association between sleep disturbances assessed at age 10 years and risk for the emergence of suicidal behaviors reported by age 12 years?

**Findings:**

In this cohort study of 8807 participants in the Adolescent Brain Cognitive Development Study, parent-reported preadolescent sleep disturbances were associated with risk for suicidal ideation and suicide attempts up to 2 years later. Adjusting for covariates, severe sleep disturbance, particularly nightmares and excessive daytime somnolence, increased the risk for suicidal behaviors.

**Meaning:**

Findings of this study suggest that sleep holds promise as a visible risk factor for study and an intervention target in youth suicide prevention.

## Teaser

This cohort study examines parent- and child-reported types and severity of sleep disruptions and their association with suicidal ideation and suicide attempts in late childhood to early adolescence.

## Introduction

Suicide represents a preventable public health problem and global disease burden, accounting for nearly 700 000 deaths annually worldwide.^1^ Suicidal behaviors far exceed this number, with more than 20 suicide attempts estimated for every death by suicide.^2^ Despite advancements in awareness and treatment, suicide rates have remained intractable over time and increased in select groups. Suicide is a leading cause of death among youths in the US, with rates doubling over the past 2 decades.^3^ Research also indicates that the majority of young people who die by suicide visit a health care professional in the year (88%) and month (42%) immediately preceding death, suggesting a missed intervention opportunity.^4^ This finding has resulted in calls to action and a coordinated strategy to delineate evidence-based risk factors for suicide among adolescents to advance prevention.^5,6^

Sleep disturbances are listed among the top 10 warning signs of suicide by the Substance Abuse and Mental Health Services Administration^7^ and have emerged as an evidence-based risk factor for suicidal behaviors even after adjusting for depressive symptoms.^8^ This finding has been replicated across studies diverse in design (cross-sectional and longitudinal), samples (clinical and nonclinical), assessment techniques (objective and subjective sleep measurements), and risk outcomes (suicidal ideation, suicide attempt, and death by suicide).^9,10,11,12,13,14,15,16,17^ Sleep difficulties associated with such risk include insomnia or nocturnal wakefulness,^11,16,17^ self-reported poor sleep quality,^9^ sleep variability (ie, irregularity in the timing of sleep schedules),^10^ and nightmares.^10,13,17^ Poor sleep stands apart from other suicide risk factors insofar as it is modifiable, nonstigmatizing, and highly treatable, underscoring its utility as an intervention target.

Systematic study of sleep in youth suicidal behaviors represents a relatively new area of inquiry despite early calls for research.^18^ Although developmental sleep changes occur in adolescence, a number of factors are associated with common sleep disturbances in timing and duration during this stage of development.^19,20^ Multiple reports suggest an association between sleep disturbance and adolescent suicide risk, but research appears limited by methodological constraints, including cross-sectional reports and frequent use of convenience samples.^21^ Longitudinal investigations of suicide risk at the population level also remain scarce, and study of preadolescent suicidal behaviors is rare.

We sought to address these gaps using the Adolescent Brain Cognitive Development (ABCD) Study.^22^ First, due to distinct infrastructural and cost barriers in population-based studies of suicide (ie, given its low base-rate occurrence), epidemiologic studies in this area are crucial yet scarce. This scarcity is especially true for prospectively designed representative samples evaluating adolescent suicidal behaviors. Second, despite initiatives to investigate the mechanisms of risk emphasizing developmental trajectories in the prevention of suicide,^23,24^ less is known regarding preadolescent risk for suicidal behavior emergence. Third, sleep indices and suicidal ideation are diagnostic features of depression and anxiety, underscoring the need to adjust for symptom severity, as emphasized in previous reports.^8,9^

We aimed to evaluate sleep disturbances in preadolescent children (aged 9 and 10 years) in association with longitudinal risk for suicidal ideation and suicide attempts at the 2-year follow-up. Based on the longitudinal association of sleep disturbances in adults with risk for suicidal behaviors, we hypothesized a longitudinal association between parent-reported sleep disturbances at baseline with incidence risk for suicidal ideation and future suicide attempts during the next 2 years (reported by parent or child), and this association would remain even after adjustment for baseline affective symptoms. We hypothesized that greater levels of nightmares would be associated with risk for suicidal behaviors in comparison with other sleep disturbances given the associations found in older samples.^8,10,13,17^

## Methods

### Participants

This cohort study used the ABCD Study dataset,^22^ which includes 11 877 children and their parents or primary caregivers (hereafter, parents) who were recruited at 21 sites across the US. Recruitment was conducted from June 2016 to October 2018, and children were enrolled at age 9 or 10 years. The ABCD Study aims to collect data for 10 years. For the present study, we used data release 4.0, which has mostly complete data from baseline to the 2-year follow up. Baseline data collection occurred from June 2016 to October 2018, and the 2-year follow up occurred from June 2018 to January 2021. Parents provided written informed consent, and children provided assent for study participation. The University of California, San Diego Institutional Review Board approved all procedures, and each site has a detailed protocol in place to address endorsed suicidal ideation or behavior. We followed the Transparent Reporting of a Multivariable Prediction Model for Individual Prognosis or Diagnosis (TRIPOD) reporting guideline.^25^

### Independent Variables Assessed at Baseline

#### Sleep Disturbance Scale for Children (SDSC)

At baseline (age 10 years), parents completed the 26-item SDSC, which is designed to assess childhood sleep disturbances, generating both a total score (ranging from 26 to 130) and 6 individual subscale scores, where higher scores indicated greater symptom severity. The 26 items are rated using a 1 to 5 Likert scale, with 1 being the least and 5 being the most disturbed. Internal consistency for the SDSC questionnaire is high (0.79), as is its test-retest reliability (*r* = 0.71).^26^ The SDSC total score has cut points based on a norming sample. Sleep disturbance is categorized according to the total score as minimal (26-35), moderate (36-46), elevated (47-51), high (52-66), and severe (67-130).^26^

The SDSC subscales include (1) Disorders of Initiating and Maintaining Sleep (DIMS), (2) Sleep-Disordered Breathing, (3) Disorders of Arousal, (4) Sleep-Wake Transition Disorders (SWTD), (5) Disorders of Excessive Somnolence (DOES), and (6) Sleep Hyperhidrosis or excessive sweating (SHY). To evaluate hypothesized associations between nightmares and suicidal behaviors, 1 SDSC item was added for proposed testing (item 21: “the child has nightmares which he/she doesn’t remember the next day”) to evaluate nightmare frequency, consistent with past research. This item was drawn from the Disorders of Arousal subscale, which comprises 3 items (ie, nightmares, sleepwalking, and night terrors). We did not have specific hypotheses about which subscale would be associated with the outcome, but we examined each for completeness.

#### Demographic Variables

At baseline, parents provided information about the child’s sex assigned at birth, race, ethnicity, and annual household income and parental educational level, marital status, and employment status. For sex assigned at birth, we used a binary factor (male or female). For race, we used a 4-category factor with Asian, Black, White, or other (including American Indian and Alaska Native as well as Native Hawaiian and Pacific Islander) and multiracial. For ethnicity, we used a binary factor (Hispanic or non-Hispanic). Race and ethnicity data were collected to examine their implications for study outcomes. For annual household income, we used a categorical factor with levels less than $50 000, between $50 000 and less than $100 000, $100 000 or more, or unknown. For parental educational level, we used the highest level reported between the parents, including less than a high school diploma, high school diploma or equivalent, some college, bachelor’s degree, postgraduate degree, or unknown. For parental marital status, we used a binary factor (married or any other status [eg, living together or separated]). For parental employment status, we used a binary factor (at least 1 parent working or not). Additionally, we considered site as a fixed effect in the models.

#### Child Behavior Checklist (CBCL) Anxiety and Depression Subscale

The CBCL^27^ parent-reported Anxiety and Depression subscale^28^ was used to assess baseline anxiety and depressive symptom severity. The subscale consists of 10 items, such as feels worthless,^28^ and generates a raw score that is converted to a T score by age and sex assigned at birth, with scores above 60 considered clinically significant. The subscale has been used widely as a validated measure of affective symptoms with good psychometric properties.^28,29^ Regarding symptom overlap, this CBCL subscale does not include items that assess suicidal ideation or sleep.

#### Family Variables 

Family history of depression was measured using the Family History Assessment Module.^30^ Each relative (parents, siblings, grandparents, aunts, and uncles) is given a yes or no assignment for having experienced depressed mood during their lifetime. We used the family history density scoring, wherein each relative is included in a total score, and family members are weighted based on genetic proximity to the youth.^31^ Scores ranged from 0 to 3, with 0 indicating no relatives with a family history and 3 indicating that all reported relatives had a history of depressed mood.

Family conflict was the youth’s report and was assessed using the Family Conflict subscale of the Family Environment Scale.^32^ The subscale is a 9-item measure, with items for fighting, anger, criticism, yelling, and loss of temper in the family. Scores ranged from 0 to 9, with 9 indicating high conflict.

The 5-item Parental Monitoring Scale^33^ assesses a child’s perception of parental monitoring of location, who the child spends time with, child disclosure, and monitoring via family dinner frequency. Scores ranged from 1 to 5, with 5 indicating higher parental monitoring behaviors. Family history, environment, and parent monitoring variables were included as covariates based on previous work in the ABCD Study dataset^34^ that identified their association with suicide risk.

### Outcome Measure Assessed at Baseline and 2-Year Follow-Up

The Kiddie Schedule for Affective Disorders and Schizophrenia (K-SADS-COMP) is a computerized, structured diagnostic interview completed by the child and parent for assessing the child’s disorders as described in the *Diagnostic and Statistical Manual of Mental Disorders, Fifth Edition*, and it has good convergent validity against clinical rating scales.^35,36^ When parent and youth reports are integrated, excellent concordance is observed.^37^ The K-SADS-COMP enabled the assessment of suicidal ideation as passive (ie, youth endorsed that they would be better off dead), nonspecific active (ie, youth endorsed a desire to die by suicide), or specific (ie, youth endorsed a method, intention, or plan) and of suicide attempt (ie, youth endorsed that they have made a suicide attempt). All risk groupings and endorsements at the 2-year follow-up were coded consistently with previous investigations. Questions pertaining to suicide risk assessment are similar to those in the Columbia–Suicide Severity Rating Scale,^38^ a gold-standard instrument for suicidal behaviors. K-SADS-COMP assesses both current (ie, past 2 weeks) and lifetime behaviors.

Since the study goal was to assess prospective development of suicidal behaviors, we excluded individuals who endorsed suicidal thoughts or behaviors at baseline. Thus, the individuals who met the criteria for lifetime endorsement at the 2-year follow-up were individuals whose thoughts and behaviors emerged after baseline (ie, incidence risk between ages 10 and 12 years). We considered any affirmative response from the parent or youth from either the past 2 weeks or the lifetime as an endorsement for each category of suicidal behavior. We assumed that outcomes (suicidal ideation and suicide attempt) were nonindependent, consistent with the Columbia–Suicide Severity Rating Scale^38,39,40^ and Centers for Disease Control and Prevention–defined suicidal behaviors,^41^ to reflect the increase in risk severity of suicide attempt. Suicidal behavior was evaluated as an ordered factor, where clinical severity of suicidal behavior was ranked in increasing order as follows: none, passive, active nonspecific, active specific, and attempt.

### Statistical Analysis

Baseline demographic characteristics by suicidal behavior category are presented in Table 1. For statistics, group comparisons are made using the Fisher exact test for count data with a simulated *P* value to test the independence of rows and columns. Two-sided *P* < .05 indicated statistical significance. Data analysis was performed from July 2023 to June 2024.

**Table 1.** Participant Characteristics

Characteristic	Participants, No. (%)					*P* value
	Suicidal behavior					
	None (n = 8044)	Passive (n = 317)	Active nonspecific (n = 258)	Active specific (n = 130)	Attempt (n = 58)	
Race^a^						
Asian	188 (2.3)	2 (0.6)	5 (1.9)	0	2 (3.4)	.001
Black	1162 (14.4)	40 (12.6)	46 (17.8)	14 (10.8)	11 (19.0)	
White	5298 (65.9)	216 (68.1)	149 (57.8)	86 (66.2)	26 (44.8)	
Other and multiracial^b^	1287 (16.0)	58 (18.3)	55 (21.3)	26 (20.0)	18 (31.0)	
Hispanic ethnicity^a^						
No	6331 (78.7)	254 (80.1)	204 (79.1)	100 (76.9)	44 (75.9)	.85
Yes	1624 (20.2)	57 (18.0)	53 (20.5)	28 (21.5)	13 (22.4)	
Age, y						
<10	7949 (98.8)	314 (99.1)	254 (98.4)	127 (97.7)	57 (98.3)	.52
≥10	98 (1.2)	3 (0.9)	4 (1.6)	3 (2.3)	1 (1.7)	
Sex assigned at birth						
Male	4160 (51.7)	154 (48.6)	116 (45.0)	58 (44.6)	20 (34.5)	.007
Female	3887 (48.3)	163 (51.4)	142 (55.0)	72 (55.4)	38 (65.5)	
Parental educational level^c^						
<High school diploma	369 (4.6)	6 (1.9)	13 (5.0)	10 (7.7)	2 (3.4)	.08
High school diploma or GED	709 (8.8)	21 (6.6)	22 (8.5)	14 (10.8)	10 (17.2)	
Some college	1951 (24.2)	86 (27.1)	73 (28.3)	34 (26.2)	17 (29.3)	
Bachelor’s degree	2119 (26.3)	95 (30.0)	65 (25.2)	32 (24.6)	13 (22.4)	
Postgraduate degree	2890 (35.9)	109 (34.4)	85 (32.9)	40 (30.8)	15 (25.9)	
Parental marital status						
Any other status^d^	2343 (29.1)	92 (29.0)	80 (31.0)	53 (40.8)	25 (43.1)	.007
Married	5648 (70.2)	225 (71.0)	176 (68.2)	77 (59.2)	31 (53.4)	
Annual household income, $						
<50 000	2011 (25.0)	81 (25.6)	85 (32.9)	37 (28.5)	20 (34.5)	.04
≥50 000 to <100 000	2127 (26.4)	85 (26.8)	63 (24.4)	38 (29.2)	17 (29.3)	
≥100 000	3273 (40.7)	124 (39.1)	88 (34.1)	48 (36.9)	13 (22.4)	
Unknown	636 (7.9)	27 (8.5)	22 (8.5)	7 (5.4)	8 (13.8)	
Sleep disturbance^e^						
Minimal	4300 (53.4)	143 (45.1)	104 (40.3)	53 (40.8)	23 (39.7)	<.001
Moderate	3004 (37.3)	121 (38.2)	113 (43.8)	55 (42.3)	24 (41.4)	
Elevated	385 (4.8)	22 (6.9)	15 (5.8)	10 (7.7)	4 (6.9)	
High	316 (3.9)	24 (7.6)	23 (8.9)	8 (6.2)	4 (6.9)	
Severe	39 (0.5)	7 (2.2)	3 (1.2)	4 (3.1)	3 (5.2)	

Abbreviation: GED, General Educational Development.

^a^ Race and ethnicity were provided by parents.

^b^ Other included American Indian and Alaska Native as well as Native Hawaiian and Pacific Islander.

^c^ Educational level refers to the highest level of education attained by the reporting parent or their partner.

^d^ Any other status included living together and separated.

^e^ Sleep disturbance was assessed with the Sleep Disturbance Scale for Children, with scores ranging from 26 to 130. Cut points from a norming sample were used to categorize sleep disturbance as minimal (26-35), moderate (36-46), elevated (47-51), high (52-66), or severe (67-130).

For the primary analysis, we conducted ordinal logistic regression using R, version 4.3.1 and the ordinal package (R Project for Statistical Computing).^42^ We fit a cumulative link model with a proportional odds assumption (link = logit) and used maximum likelihood estimates. Outcome was the 5-level, ordinal suicidal behavior risk designations (none, passive, active nonspecific, active specific, or attempt). We examined sleep disturbance as a factor variable with the categories of minimal (as the reference group), moderate, elevated, high, and severe. Anxiety and depression T scores were divided by 10, transforming the variable so that each unit increase corresponded to an increase in clinical severity by 1 SD in the reference sample. Parental monitoring, family history of depression, and family conflict were scaled to have an SD of 1 to facilitate interpretation of the model. As recommended by the ABCD Study Data Analysis, Informatics, and Resource Center,^43^ the models also included the following variables as fixed-effects factors: study site, race, ethnicity, age, sex assigned at birth, parental educational level, parental marital status, annual household income, and parental employment status. We then repeated the ordinal regression analysis for each of the 6 subscales of the SDSC and the additional item pertaining to nightmares to evaluate which aspects of sleep disturbance showed an association with risk for suicidal behavior.

### Handling Missing Data

Two hundred sixty-eight participants had missing values for at least 1 variable in the model. We used the Little test to evaluate whether missing values occurred at random, which indicated *P* < .001 of missing at random. Therefore, we imputed missing data from all independent variables via the randomForest package rfImpute function in R.

## Results

### Participant Characteristics

**Figure 1.**
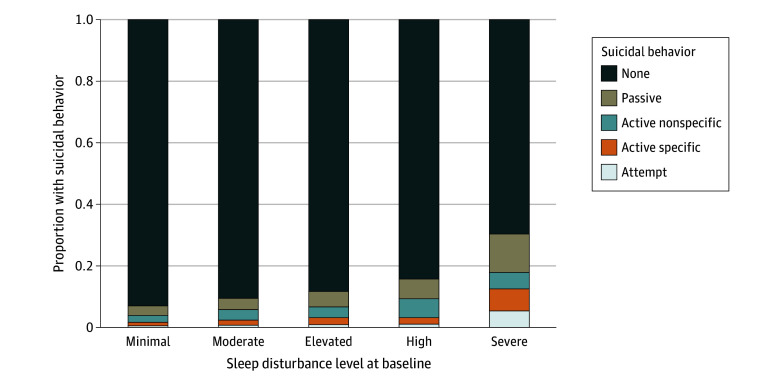
Association Between Sleep Disturbance at Age 10 Years and Suicidal Behavior at Age 12 Years The Sleep Disturbance Scale for Children, which uses cut points from a norming sample, categorized sleep disturbance as minimal (26-35), moderate (36-46), elevated (47-51), high (52-66), or severe (67-130). The stacked bar graphs depict the proportion of individuals with a given level of suicidal behavior at age 12 years based on the sleep disturbance group at age 10 years. The association between these 2 variables was tested with Fisher exact test and was significant at *P* < .001.

Of the 10 136 youths with no suicidal ideation or behavior at baseline, 8807 completed the K-SADS-COMP at 2-year follow-up and were included in the analysis (Figure 1; Table 1). At baseline, these participants had a mean (SD) age of 9.9 (0.6) years; included 4300 females (48.8%) and 4507 males (51.2%); and were identified with Asian (197 [2.2%]), Black (1273 [14.5%]), White (5775 [65.6%]), or other and multiracial (1444 [16.4%]) race and ethnicity; 1.3% had missing race and ethnicity data. The median (IQR) follow-up period was 24 (23-26) months. The final sample was categorized with the following suicidal behaviors: 8044 (91.3%) had none, 317 (3.6%) had passive suicidal ideation, 258 (2.9%) had active nonspecific suicidal ideation, 130 (1.5%) had active specific suicidal ideation, and 58 (0.7%) had a first-time suicide attempt.

### Association Between Baseline Variables and Suicidal Behavior

**Figure 2.**
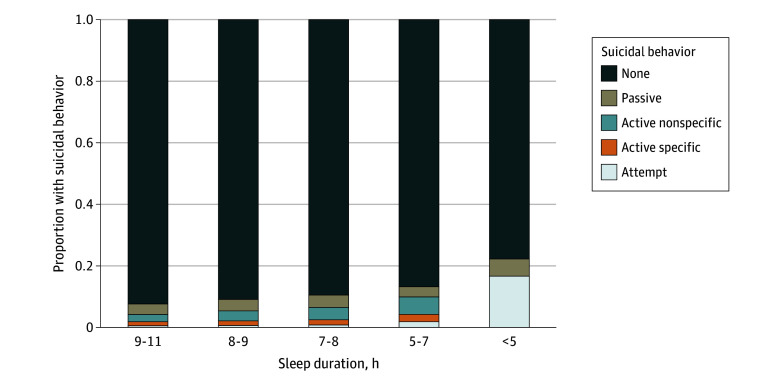
Association Between Sleep Duration at Age 10 Years and Suicidal Behavior at Age 12 Years The stacked bar graphs depict the proportion of individuals with a given level of suicidal behavior at age 12 years based on sleep duration at age 10 years. The association between these 2 variables was tested with Fisher exact test and was significant at *P* < .001.

Most participants reported few sleep problems, with 8 to 9 hours of sleep per night and 15 to 30 minutes to fall asleep being the most common responses. The primary model showed an association between baseline sleep disturbance and later suicidal behavior (X_44_^2^ = 224.6, *P* < .001; Nagelkerke *R*^2^ = 0.046), with sleep disturbance significantly contributing (X_4_^2^ = 56.1, *P* < .001) (Table 2). Relative to minimal sleep disturbance, high (odds ratio [OR], 1.39; 95% CI, 1.00-1.94; *P* = .05) and severe (OR, 2.68; 95% CI, 1.44-4.98; *P* = .002) sleep disturbances were associated with increased odds of reporting suicidal ideation or suicide attempt. Most participants reported minimal sleep disturbances at baseline (4623 [52.5%]). However, individuals with higher baseline sleep disturbance level were more likely to subsequently report suicidal behavior, and nearly 1 in 3 individuals with severe sleep disturbance later reported some level of suicidal behavior. The typical amount of sleep varied by level of suicidal behavior, with individuals who reported less sleep on a typical night being more likely to subsequently report suicidal behavior (Figure 2). At baseline, higher scores on the SDSC scale were associated with higher ratings of affective symptoms on the Anxiety and Depression subscale (ρ = 0.39; *P* < .001).

**Table 2.** Association Between Sleep Disturbance and Suicidal Behavior, Adjusting for Covariates^a^

Variable	Suicidal behavior	
	OR (95% CI)	*P* value
Sleep disturbance^b^		
Minimal	1 [Reference]	1 [Reference]
Moderate	1.17 (0.98-1.38)	.08
Elevated	1.19 (0.85-1.66)	.31
High	1.39 (1.00-1.94)	.05
Severe	2.68 (1.44-4.98)	.002
Anxiety and depression^c^	1.66 (1.46-1.89)	<.001
Family history of depression^d^	1.14 (1.06-1.22)	<.001
Family conflict^e^	1.08 (1.00-1.17)	.04
Parental monitoring^f^	0.85 (0.79-0.92)	<.001

Abbreviation: OR, odds ratio.

^a^ This model also included the following covariates as fixed effects: study site, race, ethnicity, age, sex assigned at birth, parental educational level, parental marital status, annual household income, and parental employment status. There were 8807 observations, and the Nagelkerke *R*^2^ was 0.046.

^b^ Sleep disturbance was assessed with the Sleep Disturbance Scale for Children, with scores ranging from 26 to 130. Cut points from a norming sample were used to categorize sleep disturbance as minimal (26-35), moderate (36-46), elevated (47-51), high (52-66), or severe (67-130). Minimal was the reference group.

^c^ Anxiety and depression were estimated from the Child Behavior Checklist T score for Anxiety and Depression subscale, and the raw T score was scaled by dividing by 10 so that an increase in 10 points was associated with a 1.66 increase in risk of suicidal behavior.

^d^ Family history of depression was a family history density (FHD) score, which assesses history of depression in parents, grandparents, aunts, uncles, and siblings as well as reflects the frequency of depression and the genetic proximity (eg, parents are weighted more heavily than uncles). The FHD score was *z* scored, with a mean of 0 and an SD of 1, such that an increase in 1 SD was associated with a 1.14 increased risk in suicidal behavior.

^e^ Family conflict was assessed with the youth-reported Family Environment Scale. The total score was *z* scored, with a mean of 0 and an SD of 1, such that an increase in 1 SD was associated with a 1.08 increased risk in suicidal behavior.

^f^ Parental monitoring was assessed with the Parental Monitoring Scale. The total score was *z* scored, with a mean of 0 and an SD of 1, such that an increase in 1 SD was associated with a 0.85 decreased risk in suicidal behavior.

Each increase of 10 for baseline anxiety and depression rating (eg, T score of 60 vs 50) had higher odds for suicidal behavior (OR, 1.66; 95% CI, 1.46-1.89; *P* < .001). A 1-SD increase in family history of depression density and family conflict significantly increased odds of suicidal behavior (14% increase [OR, 1.14; 95% CI, 1.06-1.22; *P* < .001] and 8% increase [OR, 1.08; 95% CI, 1.00-1.17; *P* = .04], respectively), whereas a 1-SD increase in parental monitoring was associated with a 15% reduction in odds (OR, 0.85; 95% CI, 0.79-0.92; *P* < .001) (Table 2).

Regarding demographic variables, children with other and multiracial race had higher odds of suicidal behavior than White children (OR, 1.33; 95% CI, 1.08-1.64; *P* = .007). Females had higher odds of suicidal behavior compared with males (OR, 1.43; 95% CI, 1.23-1.67; *P* < .001) (eTable 1 in Supplement 1).

### Facets of Sleep Disturbance and Suicidal Behavior

As hypothesized, the model including frequency of nightmares was significant (X_44_^2^ = 225.1, *P* < .001; Nagelkerke *R*^2^ = 0.046), with significant contribution by frequency of nightmares (X_4_^2^ = 32.0, *P* < .001) (eTable 2 in Supplement 1). Those who reported occasional nightmares at baseline (n = 118) had 74% increased odds of suicidal behavior (OR, 1.74; 95% CI, 1.07-2.85; *P* = .03) compared with those with no nightmares. Participants reporting daily nightmares at baseline (n = 8) had a 5-fold increase in odds (OR, 5.46; 95% CI, 1.42-21.04; *P* = .01) compared with those with no nightmares.

**Figure 3.**
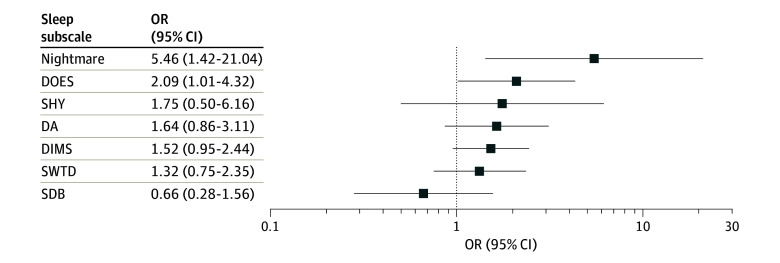
Association Between Sleep Subscales and Suicidal Behavior For all sleep subscales and nightmares, the odds ratio (OR) reflects the estimate for the most severe level of sleep disturbance relative to the minimal level. Error bars represent 95% CIs. DA indicates Disorders of Arousal; DIMS, Disorders of Initiating and Maintaining Sleep; DOES, Disorders of Excessive Somnolence; SDB, Sleep-Disordered Breathing; SHY, Sleep Hyperhidrosis; and SWTD, Sleep-Wake Transition Disorders.

Exploratory tests of subscale associations revealed the following. The subscales DIMS and DOES were associated with suicidal behavior (eTables 3-8 in Supplement 1; Figure 3). For DIMS, participants with elevated (OR, 1.40; 95% CI, 1.07-1.83; *P* = .02) or high sleep disturbance (OR, 1.40; 95% CI, 1.08-1.82; *P* = .01) compared with minimal sleep disturbance had significantly higher odds of later reporting suicidal behavior. Moderate or severe sleep disturbance showed no association. For DOES, moderate (OR, 1.30; 95% CI, 1.10-1.53; *P* = .002), high (OR, 1.66; 95% CI, 1.19-2.30; *P* = .003), and severe sleep disturbance (OR, 2.09; 95% CI, 1.01-4.32; *P* = .05) compared with minimal sleep disturbance had significantly higher odds of reporting suicidal behavior later. High SWTD (OR, 1.34; 95% CI, 1.00-1.80; *P* = .05) and SHY (OR, 1.67; 95% CI, 1.15-2.41; *P* = .007) relative to minimal had higher odds of later reporting suicidal behavior, but none of the other levels of SWTD or SHY showed an association. None of the other subscales were associated with suicidal behavior.

## Discussion

The results of this cohort study revealed that baseline sleep disturbance at age 10 years was associated with increased risk for new-onset suicidal ideation and suicide attempts by age 12 years. Specifically, high and severe sleep disturbances at baseline were associated with increased risk for suicidal behaviors in early adolescence (ie, ages 11-12 years) after adjusting for depression and anxiety, family history of depression, family conflict, and parental monitoring. Youths with severe baseline sleep disturbances demonstrated 2.7-times greater odds (OR, 2.68) for suicide ideation and suicide attempt at the 2-year follow-up.

Nightmare frequency was associated with risk for emergence of future suicidal behaviors based on a single-item assessment of nightmares. Severe nightmares, occurring daily, were associated with 5-fold increased odds of risk (OR, 5.46) for suicidal behaviors within 2 years. Regarding specific facets of sleep, excessive daytime sleepiness was associated with risk for subsequently reported suicidal behaviors, with 2.1-fold increased odds (OR, 2.09) at follow-up. Such findings show convergence with past research conducted among adult samples, as well as cross-sectional and longitudinal reports of sleep complaints and risk for suicidal behaviors, after adjusting for depression severity and affective disorders.^8,9,10,11,17^ This includes prospective investigation of poor self-reported sleep quality and objectively assessed sleep disturbances in the estimation of incidence risk for suicidal ideation, suicide attempt, and death by suicide among young adults and those in later life.^8^ Such findings align with results of preliminary sleep investigations in connection with youth suicide risk despite methodological constraints.^21^

These findings also replicated those of previous reports indicating substantial links for high family conflict and affective symptoms as well as low parental monitoring in connection with suicidal behaviors,^44^ although this study appears to be first to report on an estimation of incidence risk of suicidal behavior in the current cohort. Such findings support sleep among youths—assessed within a large longitudinal cohort—as a potential target area in the prevention of first-time suicidal behaviors. Adolescents attempt suicide at disproportionately higher rates than other age groups,^2^ which is associated with escalations in risk over time.^45,46^ The results suggest that parent-reported sleep disturbances represent a promising, visible intervention target for further research. According to at least 1 psychological autopsy study among 141 suicide decedents, sleep disturbances appear visible in the weeks and months prior to death.^12^ Specifically, adolescents were 7 times more likely to show sleep disturbances compared with community-matched controls.^12^ Although distinctly different in study design, the present study’s findings are consistent with those of other reports, supporting the assessment of sleep complaints in the presence of depression and other well-established risk factors to enhance risk detection, screening, and intervention opportunity. Based on the findings of low parental monitoring and research suggesting that earlier, parental-set bedtimes are associated with reduced suicidal risk among adolescents,^47^ additional study is recommended.

To our knowledge, this study was the first to examine sleep complaints in late childhood and preadolescent suicidal behaviors within a large, longitudinal cohort. This study addresses early prioritization and calls for research that emphasize developmental, transdiagnostic risk trajectories in the prevention of suicide,^2,23,24^ and the study of sleep and suicide prevention specifically.^48^ Adolescent sleep has been termed a perfect storm, as biological timing systems are known to change and compete with a number of factors, such as social and school demands.^19,20^ Competing demands play a role in a cascade of sleep changes and related risk for adverse outcomes as children advance through puberty and underscore the importance of healthy sleep during this unique developmental period.^20,49^ Regarding explanatory mechanisms, sleep and suicide risk cut across psychiatric disease, suggesting a shared underlying neurobiological mechanism. We propose that deficits in emotional processing may be central to this relationship^8,9,10^ based on experimental and nonexperimental manipulations of sleep and its role in cognition and emotion.^50,51,52,53^

### Limitations

Study limitations include that the measure of suicidal behavior relies on report from the parent and child rather than an objective measure. The parents may be unaware of the child’s true thoughts or actions, and the child, at age 12 years, may misunderstand the question, be uncomfortable with being truthful, or simply misremember past thoughts. Similarly, sleep information was based on parental report. Although the findings converge with past reports using polysomnography^15^ and actigraphic sleep assessments^10^ among adults, additional research using objective sleep assessments is recommended. While the sample size was large for a prospective study, the sample tended to be from families with higher socioeconomic status and living near universities; thus, the relative homogeneity of the sample may reduce the generalizability of the findings. We found associations of risk based on a single-item assessment of nightmares. Although consistent with reports showing a robust association with risk vs other sleep variables, including based on nightmare frequency alone,^8^ inventories that address nightmare frequency, intensity, and distress are recommended for future study. In addition, we modeled suicidal behavior as an ordered factor, increasing in severity, to align with the Centers for Disease Control and Prevention recommendations^41^ and for parsimony in analysis, but this approach may not accord with ideation-to-action models that posit ideation and behavior as more distinct. This study provided empirical evidence of some nonrandom association between an order in the proposed levels of suicidal behavior and sleep disturbance.

## Conclusions

In this longitudinal cohort study, greater severity of sleep disturbance at age 10 years was associated with increased risk for emergence of suicidal behaviors by age 12 years. Such findings suggest that preadolescent sleep disturbances confer near-term risk for suicidal behaviors in early adolescence, highlighting its potential importance as an intervention target. Despite evidence in older samples that sleep difficulties are prospectively associated with risk for suicide,^9^ this study appears to represent the first demonstration of this association with suicidal thoughts and behaviors in children. This is further motivated by clinical rationale insofar as sleep disturbances are overrepresented among youths, nonstigmatizing yet amenable to treatment. Poor sleep appears treatable with brief interventions for insomnia^-^and nightmares, including cognitive behavioral therapy for insomnia^54^ and imagery rehearsal treatment for nightmares,^55^ and preliminary investigation of such treatments appears promising for rapid antisuicidal response.^56,57,58^ Given its promise as a visible, nonstigmatizing, and highly treatable intervention target, we recommend additional investigation of sleep in the study and prevention of youth suicide.

## Notes

Supplement 1. **eTable 1.** Parameter Estimates of Covariates **eTable 2.** Model Results for the Nightmare Item, Adjusting for All Covariates **eTable 3.** Model Results for the DIMS (Disorders of Initiating and Maintaining Sleep) Subscale, Adjusting for All Covariates **eTable 4.** Model Results for the SDB (Sleep-Disordered Breathing) Subscale, Adjusting for All Covariates **eTable 5.** Model Results for the DA (Disorders of Arousal) Subscale, Adjusting for All Covariates **eTable 6.** Model Results for the SWTD (Sleep Wake Transition Disorders) Subscale, Adjusting for All Covariates **eTable 7.** Model Results for the DOES (Disorders of Excessive Somnolence) Subscale, Adjusting for All Covariates **eTable 8.** Model Results for the SHY (Sleep Hyperhydrosis) Subscale, Adjusting for All Covariates

Supplement 2.
Data Sharing Statement
