# Supplementary material for: Sleep Disturbance and Subsequent Suicidal Behaviors in Preadolescence
Source: JAMA Netw Open. 2024 Sep 16;7(9):e2433734. doi: 10.1001/jamanetworkopen.2024.33734 (PMC11406391; doi:10.1001/jamanetworkopen.2024.33734)
Supplement: Supplement 1. — eTable 1. Parameter Estimates of Covariates eTable 2. Model Results for the Nightmare Item, Adjusting for All Covariates eTable 3. Model Results for the DIMS (Disorders of Initiating and Maintaining Sleep) Subscale, Adjusting for All Covariates eTable 4. Model Results for the SDB (Sleep-Disordered Breathing) Subscale, Adjusting for All Covariates eTable 5. Model Results for the DA (Disorders of Arousal) Subscale, Adjusting for All Covariates eTable 6. Model Results for the SWTD (Sleep Wake Transition Disorders) Subscale, Adjusting for All Covariates eTable 7. Model Results for the DOES (Disorders of Excessive Somnolence) Subscale, Adjusting for All Covariates eTable 8. Model Results for the SHY (Sleep Hyperhydrosis) Subscale, Adjusting for All Covariates [file jamanetwopen-e2433734-s001.pdf]

Supplemental Online Content

Gowin JL, Bernert RA, Doykos TK, Sammel MD, Stoddard J. Sleep disturbance and subsequent suicidal behaviors in preadolescence. *JAMA Netw Open*. 2024;7(9):e2433734. doi:10.1001/jamanetworkopen.2024.33734

eTable 1. Parameter Estimates of Covariates

eTable 2. Model Results for the Nightmare Item, Adjusting for All Covariates

eTable 3. Model Results for the DIMS (Disorders of Initiating and Maintaining Sleep) Subscale, Adjusting for All Covariates

eTable 4. Model Results for the SDB (Sleep-Disordered Breathing) Subscale, Adjusting for All Covariates

eTable 5. Model Results for the DA (Disorders of Arousal) Subscale, Adjusting for All Covariates

eTable 6. Model Results for the SWTD (Sleep Wake Transition Disorders) Subscale, Adjusting for All Covariates

eTable 7. Model Results for the DOES (Disorders of Excessive Somnolence) Subscale, Adjusting for All Covariates

eTable 8. Model Results for the SHY (Sleep Hyperhydrosis) Subscale, Adjusting for All Covariates

This supplemental material has been provided by the authors to give readers additional information about their work.

eTable 1. Parameter Estimates of Covariates

Variables	*Odds Ratios*	*CI*	*p*
Race: Black (ref. White)	1.08	0.83 – 1.41	0.55
Race: Asian (ref. White)	0.55	0.28 – 1.06	0.08
Race: Other and Multiracial (ref. White)	1.33	1.08 – 1.64	**0.007**
Ethnicity: Hispanic (ref. non-Hispanic)	0.96	0.76 – 1.21	0.72
Age: 10+ y.o (ref. less than 10)	1.28	0.67 – 2.44	0.46
Sex: Female (ref. Male)	1.43	1.23 – 1.67	**<0.001**
Parental Education: < HS Diploma[1]	0.95	0.60 – 1.49	0.81
Parental Education: Some College[1]	1.10	0.81 – 1.48	0.55
Parental Education: Bachelor[1]	1.18	0.84 – 1.64	0.34
Parental Education: Post Graduate Degree[1]	1.15	0.82 – 1.62	0.42
Parents Married? Yes (ref. No)	0.98	0.80 – 1.19	0.80
Household Income: >=50K & <100K[2]	0.87	0.68 – 1.11	0.26
Household Income: >=100K[2]	0.82	0.63 – 1.08	0.15
Household Income: Unknown[2]	1.01	0.75 – 1.37	0.93
Parents Employed? At Least One Working[3]	1.05	0.79 – 1.39	0.73
Site: 02 (ref 01)	0.83	0.48 – 1.43	0.50
Site: 03 (ref 01)	0.51	0.29 – 0.90	**0.02**
Site: 04 (ref 01)	0.63	0.37 – 1.06	0.08
Site: 05 (ref 01)	0.67	0.36 – 1.27	0.22
Site: 06 (ref 01)	0.95	0.56 – 1.61	0.86
Site: 07 (ref 01)	0.91	0.47 – 1.75	0.78
Site: 08 (ref 01)	0.96	0.51 – 1.81	0.90
Site: 09 (ref 01)	0.82	0.46 – 1.48	0.52
Site: 10 (ref 01)	0.81	0.49 – 1.34	0.41
Site: 11 (ref 01)	0.82	0.45 – 1.48	0.50
Site: 12 (ref 01)	0.74	0.43 – 1.29	0.29
Site: 13 (ref 01)	0.79	0.47 – 1.33	0.37
Site: 14 (ref 01)	0.62	0.34 – 1.13	0.12
Site: 15 (ref 01)	0.85	0.48 – 1.50	0.58
Site: 16 (ref 01)	0.81	0.49 – 1.34	0.41
Site: 17 (ref 01)	0.74	0.42 – 1.31	0.30
Site: 18 (ref 01)	1.21	0.68 – 2.16	0.52
Site: 19 (ref 01)	0.65	0.36 – 1.14	0.14
Site: 20 (ref 01)	0.83	0.49 – 1.41	0.50
Site: 21 (ref 01)	0.79	0.46 – 1.36	0.40
Site: 22 (ref 01)	1.59	0.33 – 7.66	0.56
Observations	8807		
R^2 Nagelkerke	0.046		

1 Reference group is “High School Diploma”
2 Reference group is “<50K”
3 Reference group is “No parents working”

© 2024 Gowin JL et al. *JAMA Network Open*

eTable 2. Model Results for the Nightmare item, adjusting for all covariates

Variables	*Odds Ratios*	*CI*	*p*
Nightmare Frequency[1]: Occasionally	1.17	0.96 – 1.41	0.11
Nightmare Frequency [1]: Sometimes	1.74	1.07 – 2.85	**0.03**
Nightmare Frequency [1]: Often	0.50	0.06 – 4.03	0.51
Nightmare Frequency [1]: Daily	5.46	1.42 – 21.04	**0.01**
Observations	8807		
R^2 Nagelkerke	0.046		

1. The reference group is never

eTable 3. Model Results for the DIMS (Disorders of Initiating and Maintaining Sleep) subscale, adjusting for all covariates

Variables	*Odds Ratios*	*CI*	*p*
Sleep Disturbance[1]: Moderate	1.20	1.00 – 1.45	0.06
Sleep Disturbance[1]: Elevated	1.40	1.07 – 1.83	**0.02**
Sleep Disturbance[1]: High	1.40	1.08 – 1.82	**0.01**
Sleep Disturbance[1]: Severe	1.52	0.95 – 2.44	0.08
Observations	8807		
R^2 Nagelkerke	0.047		

1. The reference group is minimal

© 2024 Gowin JL et al. *JAMA Network Open*

eTable 4. Model Results for the SDB (Sleep-Disordered Breathing) subscale, adjusting for all covariates

Variables	*Odds Ratios*	*CI*	*p*
Sleep Disturbance[1]: Moderate	0.90	0.76 – 1.06	0.22
Sleep Disturbance[1]: High	0.98	0.73 – 1.32	0.91
Sleep Disturbance[1]: Severe	0.66	0.28 – 1.56	0.34
Observations	8807		
R^2 Nagelkerke	0.045		

1. The reference group is minimal

© 2024 Gowin JL et al. *JAMA Network Open*

eTable 5. Model Results for the DA (Disorders of Arousal) subscale, adjusting for all covariates

Variables	*Odds Ratios*	*CI*	*p*
Sleep Disturbance[1]: Moderate	1.11	0.92 – 1.34	0.29
Sleep Disturbance[1]: Elevated	1.15	0.84 – 1.57	0.38
Sleep Disturbance[1]: High	1.29	0.79 – 2.12	0.31
Sleep Disturbance[1]: Severe	1.64	0.86 – 3.11	0.13
Observations	8807		
R^2 Nagelkerke	0.047		

1. The reference group is minimal

© 2024 Gowin JL et al. *JAMA Network Open*

eTable 6. Model Results for the SWTD (Sleep Wake Transition Disorders) subscale, adjusting for all covariates

Variables	*Odds Ratios*	*CI*	*p*
Sleep Disturbance[1]: Moderate	1.06	0.89 – 1.26	0.51
Sleep Disturbance[1]: Elevated	0.97	0.73 – 1.31	0.86
Sleep Disturbance[1]: High	1.34	1.00 – 1.80	**0.05**
Sleep Disturbance[1]: Severe	1.32	0.75 – 2.35	0.34
Observations	8807		
R^2 Nagelkerke	0.047		

1. The reference group is minimal

© 2024 Gowin JL et al. *JAMA Network Open*

eTable 7. Model Results for the DOES (Disorders of Excessive Somnolence) subscale, adjusting for all covariates

Variables	*Odds Ratios*	*CI*	*p*
Sleep Disturbance[1]: Moderate	1.30	1.10 – 1.53	**0.002**
Sleep Disturbance[1]: Elevated	1.26	0.82 – 1.96	0.29
Sleep Disturbance[1]: High	1.66	1.19 – 2.30	**0.003**
Sleep Disturbance[1]: Severe	2.09	1.01 – 4.32	**0.05**
Observations	8807		
R^2 Nagelkerke	0.050		

1. The reference group is minimal

© 2024 Gowin JL et al. *JAMA Network Open*

eTable 8. Model Results for the SHY (Sleep Hyperhydrosis) subscale, adjusting for all covariates

Variables	*Odds Ratios*	*CI*	*p*
Sleep Disturbance[1]: Moderate	1.18	0.94 – 1.47	0.16
Sleep Disturbance[1]: Elevated	1.03	0.60 – 1.80	0.91
Sleep Disturbance[1]: High	1.67	1.15 – 2.41	**0.007**
Sleep Disturbance[1]: Severe	1.75	0.50 – 6.16	0.38
Observations	8807		
R^2 Nagelkerke	0.051		

1. The reference group is minimal

© 2024 Gowin JL et al. *JAMA Network Open*
