# Supplementary material for: Sleep Disturbance and Subsequent Suicidal Behaviors in Preadolescence
Source: JAMA Netw Open. 2024 Sep 16;7(9):e2433734. doi: 10.1001/jamanetworkopen.2024.33734 (PMC11406391; doi:10.1001/jamanetworkopen.2024.33734)
Supplement: Supplement 2. — Data Sharing Statement [file jamanetwopen-e2433734-s002.pdf]

Data Sharing Statement

Gowin. Sleep Disturbance and Subsequent Suicidal Behaviors in Preadolescence. *JAMA Netw Open*. Published September 16, 2024. doi:10.1001/jamanetworkopen.2024.33734

Data
Data available: No

Additional Information
Explanation for why data not available: The data are available through the ABCD study at NIH.
